# Life and times: synthesis, trafficking, and evolution of VSG

**DOI:** 10.1016/j.pt.2014.03.004

**Published:** 2014-05

**Authors:** Paul T. Manna, Cordula Boehm, Ka Fai Leung, Senthil Kumar Natesan, Mark C. Field

**Affiliations:** 1 Division of Biological Chemistry and Drug Discovery, University of Dundee, Dundee, DD1 5EH, UK; 2 Department of Pathology, University of Cambridge, Tennis Court Road, Cambridge, CB2 1QP, UK

**Keywords:** *Trypanosoma brucei*, protein sorting, exocytosis, endocytosis, protein turnover, variant surface glycoprotein, evolution

## Abstract

Evasion of the acquired immune response in African trypanosomes is principally mediated by antigenic variation, the sequential expression of distinct variant surface glycoproteins (VSGs) at extremely high density on the cell surface. Sequence diversity between VSGs facilitates escape of a subpopulation of trypanosomes from antibody-mediated killing. Significant advances have increased understanding of the mechanisms underpinning synthesis and maintenance of the VSG coat. In this review, we discuss the biosynthesis, trafficking, and turnover of VSG, emphasising those unusual mechanisms that act to maintain coat integrity and to protect against immunological attack. We also highlight new findings that suggest the presence of unique or highly divergent proteins that may offer therapeutic opportunities, as well as considering aspects of VSG biology that remain to be fully explored.

## Highlights

• Variant surface glycoprotein (VSG) is a paradigm for antigenic variation.
• VSG provides a mechanism for immune evasion.
• Rapid transport, turnover, and endocytosis contribute to VSG function.
• VSG has provided, and continues to offer, important insights into trypanosome biology.

## The variant surface glycoprotein and a paradigm for antigenic variation

For African trypanosomes that must survive in the extracellular spaces of the mammalian host, a sophisticated immune evasion mechanism, antigenic variation, is essential. We have known for over 40 years that this process is mediated by the VSG, originally described by Cross in a pioneering piece of biochemistry, and that this protein corresponds to an electron-dense coat at the surface of mammalian infective forms, as observed by Vickerman [1–3]. Both aspects remain crucial components of the modern paradigm of antigenic variation in trypanosomes.

We now understand VSG to be a 58-kDa glycophosphatidylinositol (GPI)-anchored glycoprotein, having predominantly an α-helical secondary structure, with highly variable sequences embedded at the N terminus and monoallelically expressed from a repertoire of hundreds of genes [4,5]. We also know that VSG is not the sole mechanism by which trypanosomes manipulate the immune systems of their mammalian hosts [6]. However, the basics of this system remain a potent reminder of the importance of VSG, in contributing to understanding trypanosome biology, acting as a model for antigenic variation in general, for early dissection of GPI biosynthesis (e.g., conceptually similar, albeit nonidentical, mechanisms are present in *Plasmodium* [7]), and as a model for intracellular transport studies [8].

Early work demonstrated (i) rapid addition of the GPI anchor to the VSG protein [9]; (ii) fast export to the cell surface in a manner involving a complex dance in molecular-weight changes correlating with various post-translational modifications [10]; (iii) rapid endocytosis and recycling pathways serving to carry VSG though the endomembrane system [11,12], and (iv) the realisation of just how abundant the VSG polypeptide is in terms of overall protein, estimated at 10^7^ copies and approximately 90% of cell surface polypeptides [13,14]. Other early significant observations included the demonstration of rapid VSG antibody capping, with implications for immune evasion [15], a phenomenon that required nearly 30 years to explain mechanistically and whose true value to immune evasion remains to be rigorously tested in an *in vivo* setting.

The massive dominance of the trypanosome surface by VSG implied that mechanisms for protein transport, targeting, and sorting may be distinct from mammalian cells, where most surface molecules have a *trans*-membrane domain, but it is now clear that many other trypanosomatids also rely heavily on a GPI anchor for surface molecule anchoring, suggesting broader significance for observations in *Trypanosoma brucei* [16]. In this review, we consider the molecular mechanisms that participate in the synthesis, targeting, and turnover of VSG, how these differ from canonical views of protein trafficking and modifications, and what such modifications may mean, both for VSG itself and for the trypanosome more generally, with some speculations on how the system arose. We explicitly do not deal with many recent advances in uncovering mechanisms of monoalleleic VSG expression and switching; interested readers are referred to reviews in this area (e.g., [4]).

## VSG biosynthesis and sorting through the secretory pathway

**Figure 1:**
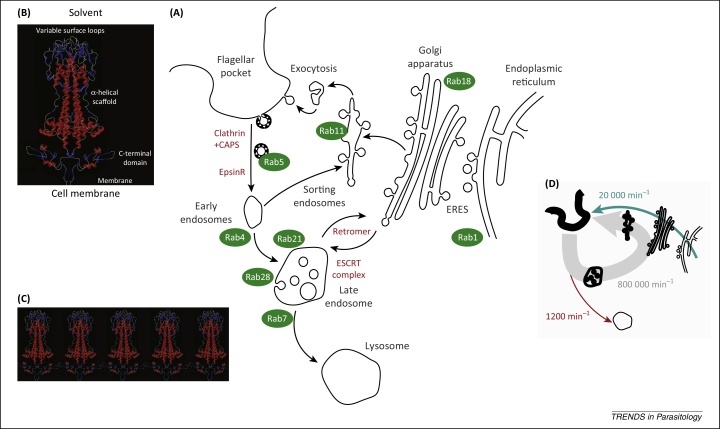
Routes responsible for trafficking of variant surface glycoproteins (VSG). **(A)** The major intracellular compartments that are known to be associated with VSG trafficking, with the flagellar pocket shown at top left. Rab GTPase proteins, which are convenient markers for many subcellular compartments, are shown as green ovals, whereas arrows indicate the major known transport routes and red indicates several molecular complexes within the endocytic system that are of significance. **(B)** The overall structure of a VSG dimer, colour coded with α-helical elements in red, β-sheets in blue, comprising the X-ray and NMR structures for the N- and C-terminal domains [83,84] (note that the arrangement of these domains with each other is arbitrary because this is not experimentally known). **(C)** A hypothetical array of VSGs with the tips of the C-terminal domains just touching. **(D)** A simplified version of (A) but rekeyed to illustrate the significant concentration gradient of VSG as it exits the ER and is then concentrated at the plasma membrane. Arrows indicate major routes, with blue for biosynthesis, red for degradation and grey for recycling. The approximate number of VSG molecules that are in flux through each route is also indicated. Abbreviation: CAPS, clathrin-associated proteins; ERES, endoplasmic reticulum exit sites; ESCRT, endosomal-sorting complex required for transport.

The bloodstream form of *T. brucei* is under stringent selective pressure from the host immune system to maintain the high-density VSG surface coat, which presumably is of sufficient density to protect against antibody recognition of invariant antigens. However, responses to experimental manipulation of VSG synthesis demonstrate an importance for VSG coat fidelity beyond simply acting as a steric barrier against the humoral immune response. VSG depletion by RNAi triggers a precise cell cycle arrest and general protein translation block, suggesting that synthesis is monitored and tightly coupled to a cell cycle checkpoint, although, because these studies were performed *in vitro*, the impact on immune evasion has not been directly addressed [17,18]. However, this phenomenon may reflect a pressing need to balance precisely the various fluxes of VSG trafficking to and from the surface, and implies the presence of a counting mechanism. Thus, efficient VSG synthesis, processing, surface delivery, and turnover are essential to satisfy demands, both intrinsic and extrinsic to the parasite, that govern its proliferation and survival (Figure 1). Although the half-life of VSG is long compared with the cell cycle, of the order of 72 h [11], the parasite still synthesises approximately 10^7^ mature copies of VSG per cell during each 8-h cell cycle [13]. This equates to approximately 20 000 VSG polypeptides per minute, all of which must be correctly processed and targeted to the cell surface [19]. Given that additional evidence suggests that VSG is overexpressed by approximately threefold during synthesis [20], the true number may approach 80 000 VSG molecules synthesised per minute, likely a significant burden on the cell. The general features of the eukaryotic membrane protein synthetic and exocytic pathways are well conserved in *T. brucei*, albeit with some idiosyncrasies, notably in glycosylation pathways and spatial organisation (Figure 1). VSG biosynthesis is outlined below, with particular attention to these adaptations and how they may aid in meeting the extreme VSG demand.

### Endoplasmic reticulum translocation

The nascent VSG polypeptide encodes an N-terminal hydrophobic signal sequence, targeting the protein to the endoplasmic reticulum (ER) lumen via the Sec61 translocon pore [21,22]. Early work suggested that this signal sequence peptide was cleaved co-translationally [21], supporting a model of classical signal recognition particle (SRP)-mediated, co-translational ER translocation [23]. More recent analyses have suggested that SRP-dependent co-translational translocation is more important for polytopic membrane proteins in *T. brucei*, with proteins destined as becoming GPI anchored by using an alternative post-translational pathway [24–26]. Given that involvement of these two pathways in VSG translocation has not been addressed directly, the exact mechanism of VSG ER import remains an open question.

### Signal sequence cleavage and glycosylation

Concomitant with translocation, nascent VSG polypeptide is exposed to ER-resident enzymes and maturation of the protein begins. The N-terminal signal sequence is cleaved [21,22], presumably by the signal peptidase complex (for a comprehensive review, see [27]). Although this complex is yet to be formally characterised in *T. brucei*, a catalytic Sec11 subunit homologue is readily identifiable by homology searches (P.T. Manna and M.C. Field, unpublished). Additionally, Asn-X-Ser/Thr consensus sites are *N*-glycosylated by oligosaccharyltransferase (OST), an eight-subunit complex in higher eukaryotes, predicted by homology searches to be represented in *T. brucei* by only three paralogous catalytic stauroporine and temperature-sensitivity protein 3 (STT3) subunits in trypanosomes. This represents a potentially extreme level of minimisation, but the precise composition of the trypanosome OST remains to be elucidated and may be more complex. *N*-linked glycosylation in *T. brucei* is central to VSG expression levels [28], with all known VSGs being *N*-glycosylated at up to three sites [29,30].

*N*-glycosylation has some unusual features in trypanosomes. Whereas most eukaryotes transfer a triantennary triglucosylated oligomannose structure, Glc_3_Man_9_GlcNAc_2_, from the dolichol-linked donor, trypanosomes are unable to produce glucosylated lipid precursors and, hence, transfer nonglucosylated glycans [29]. Additionally, whereas the STT3B transfers a triantennary oligomannose structure, Man_9_GlcNAc_2_, the STT3A subunit has preference for an unusual biantennary paucimannose glycan, Man_5_GlcNAc_2_. Direct transfer of paucimannose glycans may provide a route to paucimannose or complex glycan structures, comprising approximately half of VSG *N*-glycans, sparing lipid-linked mannose precursors required for oligomannose and, importantly, also GPI-anchor biosynthesis, a potential adaptation to the enormous VSG biosynthetic load [30–32]. Although *N*-glycosylation in *T. brucei* is perhaps the clearest departure from the classical eukaryotic pathway, a range of seemingly complementary modifications has arisen in more distal processes, such as ER quality control and further processing in the Golgi complex.

### GPI anchoring

The VSG polypeptide contains a C-terminal cleavable signal for GPI-anchor addition [22,33]. Upon translocation, the GPI signal sequence is cleaved and replaced with a preformed GPI anchor by the GPI *trans*-amidase complex comprising conserved GAA1, GPI8, and PIG-T subunits, together with trypanosomatid-specific TTA1 and TTA2 [34]. The VSG GPI anchor is unique in having only myristate (C_12:0_) fatty acids and a poly-α-galactose side chain, at least one galactose likely being transferred in the ER [35,36]. Only correctly GPI-anchored VSG is able to reach the cell surface, subject to proper folding and dimerisation, with GPI-deficient VSGs being retained in the ER before degradation [37,38].

### Folding and quality control

Intuitively, the high abundance of VSG suggests selection for efficient folding and maturation. However, considerable overproduction of VSG and subsequent proteosomal degradation of a large proportion of presumably misfolded protein does occur, suggesting that VSG is not particularly efficient in its ability to attain the native fold [20]. Although relatively little detailed mechanistic information is available, a functioning ER quality control system likely operates in VSG synthesis. The trypanosome genome encodes orthologues of many key genes for ER oligosaccharide processing and quality control, including calreticulin, the deglucosylating α-glucosidase GII enzyme and the reglucosylating UDP-Glc:glycoprotein glucosyltransferase (UGGT) [39]. Although VSG does not receive glucosylated glycans via OST, UGGT selectively binds to unglucosylated unfolded proteins and glucosylates the *N*-glycans, promoting binding to calreticulin and associated chaperones, such as BiP. VSG with glucosylated glycans is in turn a substrate for α-glucosidase GII and is further deglucosylated [39]. Once the VSG molecule has reached its native conformation, it is no longer a substrate for disorder-sensing UGGT and remains deglucosylated. Thus, freed from calreticulin, properly folded VSG is competent for ER export.

In other eukaryotes, a timing mechanism operates to free terminally misfolded proteins from this cycle and target them instead for proteosomal degradation. This involves the removal of mannosyl residues from terminally misfolded proteins to reveal a glycan structure recognised by the ER lectin Yos9, which subsequently targets the protein for proteosomal degradation [27]. *Trypanosoma brucei* encodes a cluster of ER degradation-associated mannosidase-related (EDEM) genes and a Yos9 orthologue [20,40]. Experimental support for this pathway comes from RNAi depletion of *T. brucei* EDEMs, leading to VSG accumulation [20,40]. In addition to these conserved components, two trypanosomatid-specific genes, ERAP18 and ERAP32, with positive effects on VSG copy number, were recently identified in a screen for ER residents [41]. These ER proteins have clear, but mechanistically opaque, effects upon the copy number of VSG expressed at the surface, with a potential role in quality control or ER export [41]. Together, these studies argue against VSG having superior folding competence and also demonstrate a conserved ER quality control and ER-associated degradation (ERAD) system functioning in VSG biosynthesis.

### ER export

Newly synthesised, glycosylated, and dimerised VSG is exported from the ER at specific exit sites (ERES) that, in *T. brucei*, are closely apposed to the Golgi apparatus, suggesting that they are a streamlining adaptation for high VSG flux [42]. As in other eukaryotes, transport from the ERES is via coat protein complex II (COPII) vesicles, formed following Sar1 activation and recruitment of a prebudding complex of Sec23 and Sec24, which in turn recruits the outer coat of Sec13 and Sec31 [43]. *Trypanosoma brucei* has two isoforms each of Sec23 and Sec24, with VSG export depending upon TbSec23.2 and TbSec24.1 [42]. How VSG is selectively loaded into COPII vesicles by this prebudding complex, in the absence of cytosolic sorting signals, is yet to be defined.

### Processing in the Golgi

ER-derived COPII vesicles fuse with the *cis* face of the Golgi, and cargoes undergo further processing and migrate towards the *trans-*Golgi network (TGN) for sorting to their correct cellular location. In a further example of the streamlining of the trypanosome secretory system, the Golgi is present as a single copy and occupies a defined position within the cell, adjacent to the ERES and polarised towards the flagellar pocket (FP) [44]. Within the Golgi, the α-galactose side chain of the VSG GPI anchor is extended [45,46], and *N*-glycans are further modified. It appears that only the unusual biantennary Man_5_GlcNAc_2_ glycans are processed to complex class oligosaccharides, suggested to result from the lack of a Golgi α-mannosidase and an unusual preference of trypanosome *N*-acetylglucosaminyltransferase I (GnTI) for Man_4_GlcNAc_2_ and Man_3_GlcNAc_2_ structures [47].

### Cell surface delivery

Compared with the earlier stages of VSG biosynthesis, little is known of the mechanisms of post-Golgi VSG exocytosis. Newly synthesised VSG does not enter the system for Rab11-dependent surface delivery alongside endocytosed VSG [48,49]. Golgi export of newly synthesised VSG is also actin independent [50]. In other organisms, the export of GPI-anchored proteins from the Golgi has been suggested to occur through accumulation in specific TGN microdomains, potentially involving localised lipid interactions [51], but VSG-enriched microdomains have not been observed in the *T. brucei* endomembrane system [14]. The amount of newly synthesised VSG being delivered to the cell surface at any one time is dwarfed by the constantly recycling cell surface pool, significantly complicating experimental dissection of this aspect.

### Concentration and sorting along the exocytic pathway

Newly synthesised VSG reaches the cell surface with a t_1/2_ of approximately 15 min [10], with the concentration of VSG at the cell surface approximately 50-fold greater than in the ER, and the Golgi having an intermediate density of approximately 2.7-fold that of the ER [14]. Rapid transit of VSG through the exocytic apparatus, together with a large concentration gradient, suggests efficient sorting of VSG at multiple levels [14]. The first sorting and concentration step for which we have evidence is selective export of VSG from the ER in Sec23.2/Sec24.1 COPII vesicles, and away from a population of COPII vesicles bearing the alternate paralogues Sec23.1/Sec24.2 [42]. It is calculated that a single COPII vesicle should be capable of accommodating approximately 600 VSG dimers, at a density of approximately 30 000 dimers/μm^2^, the observed density of VSG at the cell surface [13,14,19]. The observed density of VSG at the Golgi, together with the rate of synthesis, suggests one fully loaded COPII vesicle equivalent docking at the Golgi every 3.5 s. There is no apparent gradient in VSG density across the Golgi, although it is likely that this has not been measured with sufficient sensitivity to detect a moderate gradient [14], but this does suggest similar kinetics for transport between cisternae. However, substantial discrepancies exist between the observed transit rate and the theoretical rate as calculated, based on the required flux of VSG required to build the surface, indicating that there are fundamental details remaining to be uncovered [19]. Therefore, any consideration of the kinetics of VSG transit through specific exocytic organelles currently remains speculative. A second major sorting step likely occurs on exit from the Golgi, where VSG is again presumably concentrated; unfortunately, the mechanism of this transport step also remains elusive.

## Adaptations and specialisations of the endocytic machinery

The plasma membrane recycling machinery has a major role in VSG coat maintenance. All surface membrane traffic in *T. brucei* is routed via the flagellar pocket (FP), a flask-shaped invagination of the plasma membrane surrounding the base of the flagellum, where exocytosis, endocytosis and/or recycling intersect. The FP represents 2% of total surface membrane and is delineated by an electron-dense flagellar pocket collar acting as a tight cytoskeletal barrier (discussed in [8]). Uptake from the FP depends exclusively on clathrin-mediated endocytosis (CME), with inhibition of CME by depletion of clathrin resulting in severe FP enlargement, likely a result of imbalance in trafficking of material to and from the surface [52]. Endocytosis is developmentally regulated and upregulated approximately tenfold in mammalian- compared with insect-form trypanosomes, sufficient so as to internalise an area equivalent to the entire plasma membrane every 12 min [12,53,54]. Developmental regulation is mainly restricted to early endocytic gene expression as detected by transcriptome analysis, with little evidence for changes elsewhere, that is, anterograde transport or the terminal steps in endocytosis [55].

### Endocytic activity and removal from the FP

Compared with most other studied eukaryotes, the CME machinery in trypanosomes appears simplified: notably one of the CME key players, adaptor protein 2 (AP-2), a heterotetrameric complex responsible for cargo receptor binding and clathrin recruitment to the plasma membrane, is absent from all trypanosomatids that have VSG, likely a critical adaptation for rapid endocytosis and apparently unique to this lineage [56]. The absence of AP-2 also suggests that the initial endocytic step in trypanosomes, budding and internalisation of membrane from the FP, is nonconcentrative. Indeed, further concentration of VSG into endocytic vesicles may be precluded by the density of the molecule at the cell surface, with sorting of other surface molecules delegated to later steps to maximise the speed of VSG removal. It is also significant that the level of conservation of proteins interacting with clathrin appears to be low, with evidence for significantly distinct cohorts of polypeptides operating [57,58]; however, a full description of this process is lacking and, hence, the real level of divergence is unclear.

### Sorting, recycling, and return to the surface

The high rate of endocytosis necessitates a mechanism to return VSG to the surface. This process is rapid, with estimates of between approximately 1 and 10 min, and the itinerary well characterised, with dependence on Rab5 and Rab11 being clear from both morphological and kinetic studies [6,8,12,48,59]. Much of this machinery is also likely conserved; for example, recycling depends on the trypanosome orthologue of RME-8, which is essential [60], and Rab11, which interacts with the exocyst subunit Sec15, itself a well-characterised player in exocytic transport in other eukaryotes [61,62]. However, Rab11 also interacts with at least one protein restricted to trypanosomes, RBP74, and a protein restricted to taxa with motile cilia and/or flagella, AZI1, which may link Rab11 to flagellar function [62]. Rab11 is likely a major organiser of the endomembrane system, and may receive input allowing coordination of transport to balance VSG synthesis, recycling, and the requirement for protein turnover. Significantly, Rab11 and RME-8 are upregulated in the mammalian stage [54,60,63]. The mechanisms of sorting, and specifically how and where VSG is sorted away from surface, receptors, and other surface molecules remains to be explored (discussed below); recent evidence indicates considerable complexity within the endocytic apparatus, with a new compartment defined by Rab21 being added to the itinerary [64].

One fundamental question that remains is how the highly abundant VSG is sorted efficiently to a recycling pathway, while other molecules are delivered to the lysosomal pathway for degradation. The current model, supported by immunoelectron microscopy experiments, suggests a default sorting of VSG into the recycling pathway, analogous to the known route for membrane lipids and rapidly recycling cargoes, such as the transferrin receptor in mammalian systems [48]. Under this scheme, the question better asked is perhaps how other surface molecules are sorted to the retrograde pathway. Recent evidence supports a role for ubiquitylation in this process. Invariant surface glycoprotein (ISG) 65 and 75 family proteins are among the most abundant surface molecules after VSG in mammalian stages, with approximately 50 000–70 000 molecules per cell. ISGs have a *trans*-membrane domain and conserved cytoplasmic lysines that are substrates for ubiquitin modification, which act as endocytic and sorting signals [65–68]. This modification is likely recognised by the conserved endosomal-sorting complex required for transport (ESCRT) machinery [69], although the role of ESCRTs in ISG degradation has been recently challenged [70].

### Contribution to immune evasion

African trypanosomes evolved at least three VSG-dependent strategies for immune evasion. Long-term persistence is accomplished foremost by antigenic variation [71–74]. Additionally, the host immune system is weakened by general immune suppression via as yet unknown mechanisms, but which appear to compromise the ability of the antibody response to mature fully; there is evidence that fragments of VSG contribute to this [75–77]. A third mechanism involves rapid clearance of antibodies, aided by hydrodynamic flow generated by the motility of the parasite [15,78].

**Figure 2:**
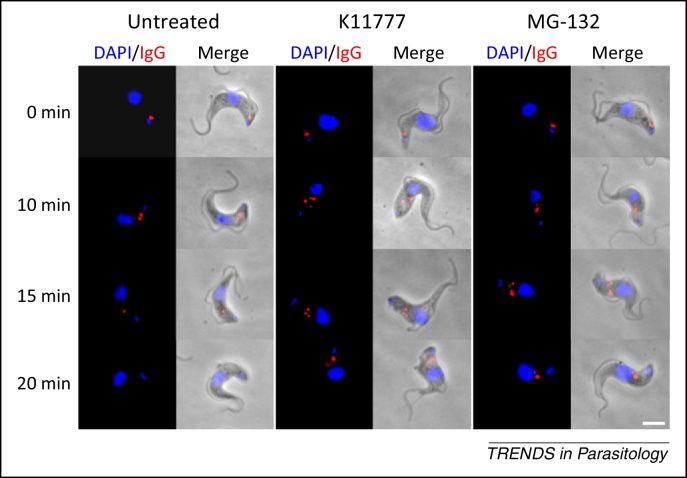
Second-line defence and degradation of antibodies. Immunofluorescence images showing degradation of anti-variant surface glycoproteins (VSG) immunoglobulin G (IgG) by bloodstream-form *Trypanosoma brucei* and the inhibition of this process by selective protease inhibitors. The internalised red dots correspond to IgG localised within the endomembrane system, whereas blue structures are the nucleus and kinetoplast (large and small, respectively). In the examples shown, the cysteine protease inhibitors K11777 and MG-132 are used to prevent the degradation of antibody so that the signal persists at the end of the time course. Scale bar = 2 um.

Given that VSG monomers are packed at high density, only the external N terminus of the homodimer is exposed to the host immune system [79,80]. Therefore, antibody-bound VSG molecules protrude from the surface coat and accumulate at the posterior pole of the cell due to increased drag, before internalisation and rapid degradation by a process requiring both the endocytic system and recycling [81]. Accumulation of VSG–immunoglobulin G (IgG) complexes at the posterior pole is independent of endocytosis, because downregulating clathrin by RNAi blocks endocytic trafficking, whereas VSG–IgG accumulation at the posterior pole is unaffected [80]. Motility is required for this phenomenon, and downregulation of the dynein arm intermediate chain DNAI1, which reverses the swimming direction of the parasite, results in accumulation of IgG at the anterior pole of the cell [80]. Immunoglobulin degradation is sensitive to E46d and K11777 (cathepsin and cysteine protease inhibitors, respectively), supporting a role for lysosomes in degradation (Figure 2), whereas VSG is efficiently recycled back to the surface [12,48,81].

Although the precise role of surface antibody clearance in the mammalian host is yet to be fully elucidated, its effectiveness is evident at low to moderate antibody concentrations, but clearly insufficient to mediate protection at high antibody titres [82]. Thus, this represents a mechanism that protects an individual cell during emergence of a specific humoral immune response, and may also be of importance during early infection, when antibody titres are low or after differentiation to the cell cycle-arrested short stumpy form.

## Evolution of the VSG surface

**Figure 3:**
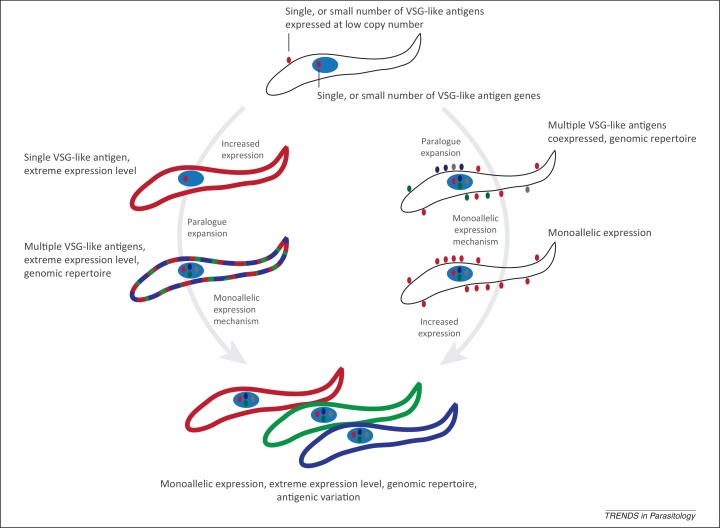
Steps in the generation of an immune evasion machine. To evolve the variant surface glycoproteins (VSG) system, it is likely that the ancestor of the modern VSG-expressing trypanosomes expressed a single or small family of surface proteins, similar to the gp63 of *Leishmania* and that is present throughout the kinetoplastids. Two possible subsequent routes can be envisaged, but in both, paralogous expansion must have preceded the evolution of a monoallelic expression mechanism. On the right is a model where paralogous expansion of the proto-VSG family occurred before the evolution of a monoallelic expression system, and before extreme expression levels becoming prevalent. The early steps of this pathway are similar to the situation in *Trypanosoma cruzi*, where an extensive family of mucins and other proteins is expressed, but where there is no evidence for monoallelic expression. A second model suggests that high levels of expression arose first, and this was followed by paralogous expansion, so that a complex, dense coat was formed, which contained more than one proto-VSG. Addition of a monoallelic expression system then produced the situation seen in African trypanosomes.

How then did VSG arise, and with it some of the remarkable aspects of trypanosome cell biology? We can presume that *T. brucei* initially arose from an organism more similar to *Leishmania* and *Trypanosoma cruzi*, which implies a more heterogenous surface proteome than African trypanosomes currently have. Massive increases in expression level, expansion into a large paralogous gene family, and the advent of monoallelic expression were all required to achieve the VSG system, but the order of events is unclear (Figure 3). Comparisons of the gene complements and genome structures between the African and American trypanosomes, and *Leishmania* spp*.* indicate that the major coding variance resides within those genes encoding surface determinants, with a remarkable level of conservation for most other gene functional classes, even extending to the level of retaining synteny and polycistronic transcription unit composition [85]. This insight agrees well with much of the earlier biochemical analysis demonstrating clear diversification of the surface composition of the African and American trypanosomes and *Leishmania* spp, and is presumably a reflection of the major emphasis of selective pressure for parasites with radically different life cycles and styles [86].

Examination of more closely related genomes also suggests how VSG itself has evolved, with clear evidence for ongoing and lineage-specific diversification of this family. For example, the VSG repertoire of *Trypanosoma vivax* is greater than *T. brucei* in terms of sequence diversification, with evidence for differential recombination events between taxa and little overlap between the repertoires. A deep division into two distinct subfamilies, a-VSG and b-VSG, appears to have been maintained through the evolution of the African lineage, suggesting that isolation between these two subfamilies occurred early [87,88]. Furthermore, VSGs also appear to have given rise to nonvariable antigens, such as the transferrin receptor and serum resistance associated (SRA), and overall surface diversity may also be connected with the expression site (ES) architecture. For example, it has been suggested that ISGs are related to ESAG11, providing additional possible mechanisms for surface phylum diversification and separation of the evolutionary trajectories of surface proteins encoded by core chromosomal regions and those that have migrated to subtelomeric sites [88]. *Trypanosoma grayi*, also a tsetse-transmitted trypanosome of African origin and closely relate to *T. cruzi* [89], lacks VSG, which suggests that emergence of the VSG system was rapid, because no intermediate forms between *T. grayi* and the ‘*T. brucei* African clade’ are known [56].

## Concluding remarks

VSG has played the role of a prototypical model protein for understanding the cell biology of the GPI anchor and the molecular genetics and immunology of antigenic variation, making it an exceptionally generous molecule, if at the same time sitting at the heart of a deadly immune evasion machine. Moreover, interest in just these topics propelled the emergence of African trypanosomes as an important model organism, so that understanding of the cell biology of VSG is now at an advanced stage, albeit with many questions that still remain. Without the initial spur provided by VSG, it is doubtful that the interest or research activity into African trypanosomes would be as strong or as vibrant as it is today, and many of us have benefited from the generosity of VSG. VSG continues to challenge and fascinate; however, a full appreciation of this remarkable example of evolution and immune evasion machinery remains to be achieved.
